# Polymer-Based Bone Substitutes in Periodontal Infrabony Defects: A Systematic Evaluation of Clinical Studies

**DOI:** 10.3390/polym13244445

**Published:** 2021-12-18

**Authors:** Florin Onisor, Simion Bran, Ileana Mitre, Alexandru Mester, Andrada Voina-Tonea, Gabriel Armencea, Mihaela Baciut

**Affiliations:** 1 Department of Maxillofacial Surgery and Implantology, University of Medicine and Pharmacy, Iuliu Hatieganu, 400012 Cluj-Napoca, Romania; florin.onisor@gmail.com (F.O.); ileana.mitre@umfcluj.ro (I.M.); mbaciut@yahoo.com (M.B.); 2 Department of Oral Health, University of Medicine and Pharmacy, Iuliu Hatieganu, 400012 Cluj-Napoca, Romania; 3 Department of Dental Materials, University of Medicine and Pharmacy, Iuliu Hatieganu, 400012 Cluj-Napoca, Romania

**Keywords:** polymer, bone substitute, infrabony defect, periodontal regeneration

## Abstract

*Background and Objectives:* The aim was to systematically review the available literature regarding the use of polymers as a bone substitute for the treatment of periodontal infrabony defect. *Materials and methods:* Three databases (PubMed, Scopus and Web of Science) were searched to find all relevant studies published in English from inception until September 2021 using a combination of keywords. The inclusion criteria consisted of human clinical studies which reported the use of a polymer-based bone substitute in the treatment of infrabony defects. *Results:* 164 studies were provided from the databases. Of these, five articles were eligible and reported favorable outcome in terms of probing depth, clinical attachment gain and defect fill at the follow-up (3 months and 6 months). *Conclusions:* Polymer based-bone substitutes may represent a useful alternative in treating infrabony defects. Due to the limited number of studies, more research is needed to sustain the advantages of these products.

## 1. Introduction

Periodontal disease represents a chronic inflammatory disease which affects periodontal structures (such as gingiva, cementum, periodontal ligament and bone) that results in the loss of periodontal attachment [1,2]. The loss of periodontal structures has been characterized by the spread of bacterial biofilm along the root surface of teeth. Besides, bacterial inoculation, it is worth mentioning other genetic and epigenetic factors such as age, sex, alcohol and tobacco consumption and the presence of systematic disease (e.g., diabetes, cardiovascular disease, hepatitis, cancer) [1,2,3]. When it comes to infrabony defects, defects may be found in the interalveolar and marginal bone. Infrabony defects are a consequence of periodontal disease due to local factors (e.g., plaque accumulation, food debridment, tooth position, occlusal trauma) [4].

Deep periodontal pockets, with their bases exposing the alveolar bone can also benefit of treatment approaches that involve biomaterials [5]. Infrabony defects represent a class of alveolar bone deficiencies and can have one, two or three osseous walls. They are often associated with the presence of deep periodontal defects, even after specific therapeutic measures [5]. Given this conditions, guided bone regeneration (GBR) and guided tissue regeneration (GTR) have become valuable treatment options in periodontology. Both methods follow the activity of cellular differentiation and growth, succeeded by the induction and guidance of tissue development [6]. 

Nowadays, biomaterials are widely used in the field of craniofacial bone regeneration, given the fact that a big number of pathologies can appear or develop in this area. From different types of tumors and inflammatory processes, to congenital osseous defects, bone atrophy or periapical processes, all require surgical interventions and can induce various bone defects [7,8,9].

Along with autogenous, allogenic and xenogeneic bone grafts, but also along with synthetic bioceramics and composites, synthetic polymers are important elements in the domain of bone replacement. Imperative and precise conditions must be fulfilled by all biomaterials involved in the process of osseous regeneration [7,8]. Aspects like osteoconductivity, osteoinductivity, biodegradability, biocompatibility, mechanical and surface characteristics, handling, certification of manufacturing processes, antigenicity and degree of porosity must concur in the final biomaterial product and represent the fundament of a successfully conducted osseointegration [8]. The most frequently used polymers are represented by aliphatic polyesters which include also their subclasses, such as polyglycolic and polylactic acids, poly (ε-caprolactone) and their resultant, derivate products [7]. These products have the advantage that their manufacture can be controlled and tailored in terms of physico-chemical properties, structure, porosity, biodegradation and shape and size of the particles according to the need of treatment [8].

Nevertheless, resembling any other biomaterial used in dental medicine, every different type has its own advantages and limitations. These must be taken into consideration, alongside the specific conditions of each particular clinical case. With regards to the last statements, the aim of our article was to highlight the available clinical studies that used polymers as a bone substitute for the treatment of periodontal infrabony defect. 

## 2. Materials and Methods

This review was conducted following the guidelines of the Preferred Reporting Items for Systematic Review and Meta-Analysis (PRISMA) [10]. The research question was: “What is the effectiveness of polymer-based bone substitute in the treatment of infrabony defects?”. 

P: Patients with infrabony defects

I: Open flap debridement and the use of polymer-based bone substitute (polylactic acid, polyglycolic acid or poly ε-caprolactone)

C: Open flap debridement alone 

O: Regenerative potential of polymer based-bone substitute in infrabony defects (clinical assessment: probing depth, clinical attachment loss, bleeding on probing, defect fill)

### 2.1. Eligibility Criteria

The inclusion criteria consisted in human clinical trials or case studies, published in the English language, which reported the use of polymer-based bone substitute for infrabony periodontal defects. The exclusion criteria were the followings: case reports, letters to editors, unpublished, incomplete data or conference papers.

### 2.2. Literature Search

Three databases (PubMed, Scopus and Web of Science) were searched to find all relevant studies published in English from inception until September 2021. A combination of keywords was used: ‘‘polylactic acid’’ OR “polyglycolic acid” OR “poly ε-caprolactone” AND “intrabony defect” OR “infrabony defect”. Titles and abstracts of the papers identified were assessed for eligibility and irrelevant articles were excluded. Full-text articles previously obtained were read and assessed in order to correspond to the inclusion criteria. 

### 2.3. Data Extraction

The following data were extracted using a standard data form: first author, year of study, country, type of study, characteristics of the patients, age, periodontal measurements, results before and after the treatment and conclusions. 

### 2.4. Statistical Analysis

The main goal of our paper was to obtain a meta-analysis. Due to heterogeneity of patients’ characteristics and assessment of infrabony defect, statistical analysis could not be made.

### 2.5. Risk of Bias

In order to assess the risk of bias, The Strengthening the Reporting of Observational studies in Epidemiology Statement (STROBE) [11] was used which consisted in seven criteria composed of study design, participants, sample size, variables, potential confounders outcomes and statistical analysis. Each criterion was quantified with “1” or “0” (with a maximum achievement of “7”), if criteria was described in the included article. After the score was summed, quality of the study was included in the following threshold: low (score 0–3); acceptable (score 4–5); high (score 6–7). Included studies were analyzed by two authors and in case of disagreements, a third one was involved.

## 3. Results

### 3.1. Search Results 

**Figure 1:**
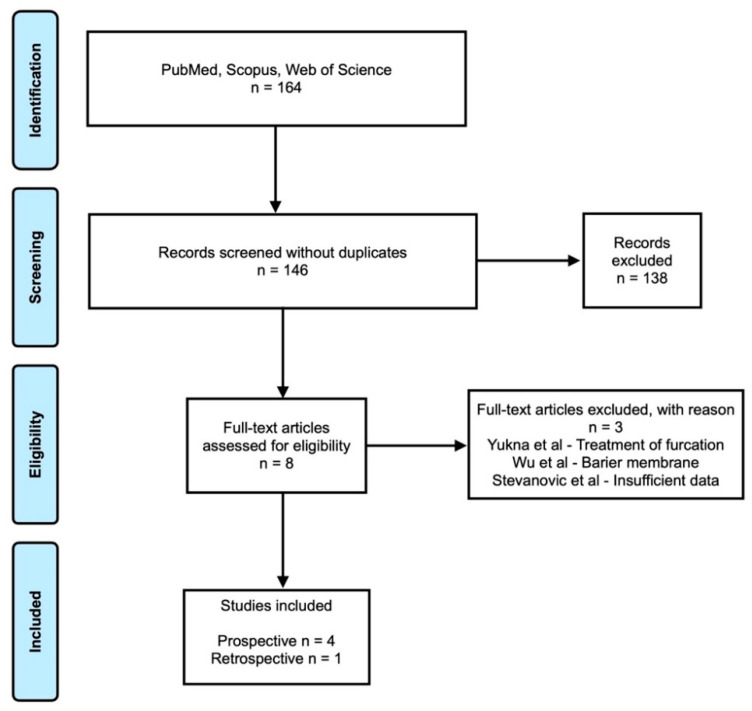
PRISMA flowchart.

A total number of 164 studies was provided from those three databases. After excluding the duplicates, a total of 146 articles were screened for eligibility. The articles were reviewed by title and abstract; eight articles were identified for full-text analyses. Of these, five articles met the inclusion criteria [12,13,14,15,16]. Reasons of the exclusion [17,18,19] are shown in Figure 1. 

### 3.2. General Characteristics 

From the retrieved articles, five studies met the inclusion criteria and were published between 1990 and 2019. Regarding the type of article, four articles are prospective clinical studies [12,13,14,15] and one articles is a retrospective case study [16]. The studies were conducted in USA [12,13,16] and India [14,15]. The sample size varied from 5 up to 40 patients. The mean age of the patients was included in all studies and varied from 28 to 58 years. Only 4 studies reported the gender of the patients.

### 3.3. Clinical Assesment of Periodontal Disease and Infrabony Defects

In the article of Yukna [12], patients included in the study were diagnosed with moderate, deep infrabony defect (≥2 mm with one wall, two walls, three walls or combination type). With regards to periodontal measurements, for each patient was assessed O’Leary index, probing depth (PD), clinical attachment gains and defect fill was calculated based on the intraoral radiographs. Before surgery, all patients received oral hygiene instruction, scaling and root planning (SRP) with polishing, occlusal adjustments and if needed teeth were splinted. Infrabony defect surgery was initiated when O’Leary index score was ≥80% positive and gingival tissues was satisfactory. 

Patients from the article Meadows and coworkers [13] were diagnosed with moderate to severe periodontal disease and each patient had a minimum of three infrabony defects. Periodontal assessment consisted in modified O’Leary index, bleeding on probing (BOP), PD, tooth mobility and defect fill was calculated based on the intraoral radiographs. As in the previous study, before surgery, each participant received an initial therapy; surgery was done when plaque index was 10%.

Prakash and coworkers [14] included patients with chronic periodontitis, with at least two infrabony defects (≥6 mm). Periodontal assessment consisted in plaque index, gingival index, PD, clinical attachment level (CAL), gingival margin and defect fill was calculated based on the intraoral radiographs. The pre-surgical procedure consisted in detailed oral hygiene instructions and plaque control with phase I periodontal therapy. Two weeks after phase I therapy, if oral hygiene and periodontal tissues responded well according to plaque and gingival index, then, surgery was considered. 

In the study of Chhabra et al. [15], patients had two- or three-wall infrabony defects. Periodontal assessment consisted in PD, CAL and defect fill was calculated based on the intraoral radiographs. All patients received oral prophylaxis, occlusal adjustments and home care instructions. If oral hygiene status had a satisfactory level, then surgery was considered.

Verandi and collaborators [16] included patients with chronic periodontitis, with at least 1 infrabony defect with 2/3 walls with an infrabony component ≥ 3 mm and PD ≥ 6 mm. Each patient received initial periodontal therapy which consisted in oral hygiene instructions SRP and re-evaluation at 6 months. Periodontal assessment consisted in full mouth plaque score, PD, CAL defect fill was calculated based on the intraoral radiographs. Surgery was considered if full mouth plaque score was <30%.

### 3.4. Treatment of Infrabony Defect with Polymer Based Bone Substitute

Yukna et al. [12] performed an open flap debridement with hand instruments and ultrasonic debridement which the experimental group received polymer-based bone substitute and control group only surgical debridement. Clinical response of experimental group showed positive results in terms of PD and clinical attachment gain (Table 1). Defect fill was higher in experimental group (2.2 mm; 60.8%) compared to control group (1 mm; 32.2%) at 6 months post-surgery. Meadows et al. [13] treated the infrabony defects using three methods: FPD alone, FPD + PLA and FPD + DFDBA. At 6 months post-surgery, all sites healed with no complications. DFDBA showed the greatest results which consisted in defect fill of 3 mm (65%) compared to FPD 0.4 mm (11.2%) and to PLA 0.1 mm (2.2%). Furthermore, probing depth decreased with 4.15 mm for DFDBA, 3.4 mm for FPD and 1.8 mm for PLA. 

Prakash et al. [9] obtained better results for OFD associated with polymer bone substitute compared to OFD alone. Mean difference between control and experimental group baseline, 3 months and 6 months are in terms of PD (0.75 mm; 1.75 mm; 1.38 mm), CAL (0.62 mm; 1.37 mm; 1.38 mm) and in terms of defect fill at 6 months was 17.3%. Chhabra et al. [10] divided patients in two groups: group A treated with polylactic-polyglycolic acids alone; group B treated with polylactic-polyglycolic acids in conjunction with polyglactin 910 membrane. Both groups showed reduction in PD, gain in CAL, and bone fill at 3 months and 6 months, respectively. A comparative evaluation of both groups at 3–6 months showed similar results in terms of PD, gain in CAL and bone fill. Veradi et al. [16] treated infrabony defects with NHA in combination with PLGA copolymer. At 12 months post-surgery showed reduction of PD, gain of CAL and defect fill compared to baseline. 

From the included studies, could be seen that the use of polymer-based bone substitute with OFD was superior to OFD alone in terms of PD, CAL % defect fill. When PLA bone substitute was compared with DFDBA, results were in favor for latter bone substitute in terms of osseous defect fill. In comparison between copolymerized polylactic-polyglycolic acids alone and copolymerized polylactic-polyglycolic acids in conjunction with polyglactin 910 membrane, both methods achieved similar results in terms of PD, CAL and defect fill.

### 3.5. Quality of the Included Studies

The results obtained according to STROBE criteria are presented in Table 2. The quality of the included papers was good, with a score which varied from 5 to 7.

## 4. Discussion

Polymer-based bone substitutes have demonstrated that they are able to provide new bone formation in case of infrabony defects. Given the fact that polymers have their individual indications, advantages and limitations, each category must be taken into consideration. 

Polylactic acid is an aliphatic polyester with complete biodegradable properties, which over the time permits an absolute osseous substitution [20]. As an advantage, this product induces a healing process without any external contamination. It can be used by itself or in combination with other products [21]. Nevertheless, its disadvantages are represented by extended degradation periods, in addition to inflammation responses of the host, caused by the elevated degrees of crystallinity in the material’s fragments [22]. Studies have shown that polylactic acid, used as a biodegradable barrier in infrabony defects improves periodontal parameters (probing measurements, attachment growth, minimization of osseous defects or volume modifications). Furthermore, in comparison with non-absorbable barriers in guided tissue regeneration, no important discrepancies were observed [23]. In cases where polymers have been used as bone substitutes, bone formation has been achieved, but with lower results in comparison with DFDBA or OFD alone [13]. In contrast, Prakash et al. mentioned that alloplastic graft was superior that OFD alone [14].

Polyglycolic acids are synthetic polymers, used for medical purposes, due to their advantages, such as biodegradation ability, optimal mechanical and thermal characteristics [24]. Research showed that polyglycolic and polylactic acid that underwent a copolymerization process can be used in the technique of guided tissue regeneration, in periodontal pathologies [15]. Furthermore, the two copolymerized acids are able to behave as a protective element in place [15]. Other studies proved that a fusion between nanohydroxyapatite in form of powder and the copolymer polylactic and polyglycolic acid may function as a treatment in infrabony defects and even offer valid outcomes, like improved attachment degree, probing parameters and radiologic osseous aspect, after one year examination, suggesting that these results could give rise to more converged investigations in the field of infrabony defects treatment [16]. Other investigations conducted a comparison between polylactic and polyglycolic acid copolymer and a collagen resorbable material and Bio-Oss, used in infrabony defects. Results demonstrated that there is no important distinction between the clinical outcomes of the two alternatives, after a one-year follow-up [25].

Polycaprolactone represents another class of synthetic polymers, that exhibits properties suitable for its use in the periodontal treatment of osseous defects [26]. It is biocompatible, degradable, and can be processed into easy-to-use bone substitute and membrane. It has the advantage to produce less immunological responses of the host and manifests no toxicity [26]. Studies showed that the use of polycaprolactone in combination with other products may amplify its efficiency in the treatment of periodontal defects [26]. A progress in osseous development was established while using polycaprolactone as a membrane in guided tissue regeneration [26]. However, this material has its own limitations, related to its hydrophobicity and the consecutive poor cell adhesion. Research indicated that alternatives like poly (ε-caprolactone) covered with graphene oxide may resolve this disadvantage, by facilitating cellular adhesion and development [27]. Other studies used bioactive glasses in combination with polycaprolactone. The bioactive glass was treated with imbandronate, a medication used in the prevention of osteoporosis, in order to intensify the osteogenesis potential of the final product. The results proved exceptional biological compatibility and elevated osteogenesis potential, in comparison to polycaprolactone on its own [28].

Current research proves that with the help of various development technologies, synthetic polymers can be adjusted, in order to obtain optimal properties [29]. Furthermore, the combination between synthetic polymers and other materials and substances often improves their characteristics and expand their field of use. A contribution to the development of this materials is represented by the fabrication technologies. Processes like CAD/CAM, tridimensional imprinting or electrostatic fiber spinning, managed to enlarge the range of applications [29]. 

Haugen and coworkers mentioned in their paper written for European Workshop that autogenous bone graft still remains the gold standard for repair of bone defects [30]. It is well known that autografts have specific and essential components which permits them to establish osteoinduction, osteoconduction and osteogenesis [31]. However, in the treatment of infrabony defects it is difficult to use a second surgical site to harvest autogenous bone due to complications like injury, deformity, scaring of the donor site, morbidity, inflammation, infection and last but not least higher expenses for the patient [30]. When it comes to compare autogenous bone with alloplastic bone grafts, latter offers multiple advantages such as the absence of potential infection transmission, ethical, religious concerns [32]. Furthermore, it should be noted that these products can be tailored in different shapes according the infrabony morphology. Fukumba and coworkers, mentioned in their paper that alloplastic bone grafts can be combined with growth factors and cell transplantation. This fact may offer the so needed properties of polymers-based bone substitute like osteoconduction, osteoinduction and osteogenesis and the concerns stated by Haugen [30] about cell attachment, pH, time of absorption and the release of acidic degradation products may be attenuated. 

Attempts to obtain an ideal bone graft of in our case polymer bone substitute is already in development. To have an ideal bone graft, the product needs to meet several criteria’s such as interconnected porosity with adequate diameter of pore bone in order to allow a connection to form with bone cells, nutrients and exchange of internal products (e.g., waste) [30]. Other requirements that need to be taken into account are vascular ingrowth, bone cell attachment, mechanical strength with a controlled biodegradability and dimensional stability [8,30]. Few studies about polymers have addressed the issues of degradability and mechanical stability of polymers. Zhao and collaborators have mentioned in their comprehensive review where they searched several databases that modifications to polymer by adding hydroxyapatite or tricalcium phosphate offer benefits in terms of bone regeneration [32]. Other researches showed that adding VEGF using a silk fibroin coating onto polymer bone substitute achieved a controlled release delivery of bioactive molecules, enhanced angiogenic properties [33,34,35]. Guduric and coworkers demonstrated that 3D printed polylactic acid-based biopolymer processed with a diameter pore of 200 μm improved cell proliferation and differentiation [36]. Another polymer that was studied and showed promising results is called poly (propylene fumarate), which demonstrated great resistance to compressive stress and a controlled biodegradability [30,37]. Besides these major advantages, their degradation led to the release of acid products which may influence negatively the native bone [37]. 

In the last decade, new research has tried to show the efficacy of using other products like platelet rich fibrin (PRF) or other biological mediators in combination with bone grafts in promoting periodontal regeneration [4,38]. Current evidence has showed that combination of inorganic bovine bone and PRF determined non-inferior results regarding CAL gain and radiographic bone level gain when it was compared with the combination of collagen membrane and bovine bone [4]. Rexhepi and coworkers are suggesting in their RCT that anatomy of infrabony defect is very important when it comes to choosing the right surgical technique; the number of bony walls and the width and the angle of root to bone surface represents the most important factors that needs to be taken into account [d]. If we are to compare the results of this current review with other research, it is difficult because there are heterogenous data among the included studies. Thus far, there’s not a clear conclusion which technique is the best in treating infrabony defects. It is well known that synthetic material cannot be used in EU countries due to regulation; because few comparative studies are available the benefits of using polymers with regards to other bone grafts are not well documented. 

The major strengths of our review were the systematic search from the databases and the methodological assessment of the included studies. Moreover, it is the first study that collects and describes data regarding the use of polymers bone grafts in infrabony defects. Nevertheless, the absence of randomized clinical trials in which polymers were used, makes these data a preliminary assessment from the literature. Our research has several limitations. One of them is the small number of patients with treated using polymers bone grafts. Further, the diagnosis of periodontal disease was not presented accurate in the included studies. Other limitations were represented by the assessment of the periodontal parameters, which was not explicit and as a repercussion the reproducibility of the measurements may not be accurate. Furthermore, the follow-up period after the periodontal surgery was not similar on all studies. What should Further studies (e.g., randomized clinical trials) are necessary to evaluate the efficiency of polymer-based bone substitute in infrabony defects. Definitely, Fukuba et al.’s statement should be taken into account that alloplastic bone grafts may be the first choice in treating bone defects only when they achieve robust capabilities [32]. 

## 5. Conclusions

Current findings have shown that there is a growing demand for development of more efficient grafting materials. The research studies in tissue engineering look to develop polymers with growth factors or with osteogenic cells. As a result of this continuous development, natural bone grafts are replaced by synthetic bone. Polymer based-bone substitute may represent a useful alternative in treating infrabony defects. Despite these progresses, several issues are needed to be fixed in order to achieve a great polymer in terms of surface structure, mechanical stability and biodegradability for new bone formation; these properties will improve the osteoinduction capacity of polymer bone. To draw a clear statement about the benefits of using polymer bone, more research is needed.

## Figures and Tables

**Table 1:** Characteristics of the included articles.

Author. Year. Country	Study Type	Patients’ Characteristics	Measurements	Results		Conclusion
				Pre-Operative	Post-Operative	
Yukna. 1990. USA [12]	Prospective	*n* = 21 Male: 14 Female: 7 Age: 29–69 years (mean age 40.6 years) Control: OFD Experimental: polymer bone	O’Leary index PD Periapical radiographs	(a) PD (mm) Control: 6.00 ± 1.3 Experimental: 6.4 ± 1.3	(a) PD (mm)–6 months Control: 3.7 ± 0.6 Experimental: 3.3 ± 0.8 (b) Gain clinical attachment (mm)–6 months Control: 1.0 ± 0.9 Experimental: 1.9 ± 1.1 (c) % Defect fill–6 months Control: 9% Experimental: 76%	Polymer bone substitute showed better results for the repair of periodontal osseous defect compared to OFD.
Meadows. 1993. USA [13]	Prospective	*n* = 10 Male: 6 Female: 4 Age: 28–58 years (mean age 42 years)	Modified O’Leary index BOP PD Defect depth Tooth mobility Periapical radiographs Photomicrographs	(a) PD (mm) FPD: 6.30 ± 1.70 DFDBA: 7.45 ± 2.25 PLA: 6.85 ± 1.63 (b) Defect depth (mm) FPD: 3.55 ± 1.34 DFDBA: 4.65 ± 0.94 PLA: 4.50 ± 1.43	(a) PD (mm)–6 months FPD: 2.90 ± 1.28 DFDBA: 3.30 ± 1.13 PLA: 5.05 ± 1.72 (b) Defect depth (mm)–6 months FPD: 1.85 ± 1.65 DFDBA: 1.05 ± 0.82 PLA: 2.85 1.31 (c) % Defect fill–6 months FPD: 11.2% DFDBA: 65% PLA: 2.2%	DFDBA showed the greatest amount of osseous defect fill. PLA did not show comparable results compared to FPD.
Prakash. 2010. India [14]	Prospective	*n* = 5 Age: 33- 55 years Control: OFD Experimental: polymer bone	Plaque index Gingival index PD CAL Periapical radiographs	(a) PD (mm) Control: 7.13 ± 1.25 Experimental: 7.88 ± 1.46 (b) CAL (mm) Control: 7.38 ± 2.33 Experimental: 8.00 ± 1.41 (c) Initial defect fill Control: 14.10 ± 5.0 Experimental: 18.56 ± 7.95	(a) PD (mm)–3 months Control: 5.38 ± 0.92 Experimental: 4.38 ± 1.06 PD (mm)–6 months Control: 5.38 ± 0.74 Experimental: 4.75 ± 0.89 (b) CAL (mm)–3 months Control: 6.50 ± 2.27 Experimental: 5.75 ± 1.28 CAL (mm)–6 months Control: 6.00 ± 2.20 Experimental: 5.50 ± 1.20 (c) Defect fill–6 months Control: 11.30 ± 7.7 Experimental: 9.50 ± 4.80 % Defect fill–6 months Control: 26.7% Experimental: 44%	Polymer bone substitute showed better results than OFD in terms of both clinical and radiographic analysis.
Chhabra. 2011. India [15]	Prospective	*n* = 40 Group A: copolymerized polylactic-polyglycolic acids alone Group B: copolymerized polylactic-polyglycolic acids in conjunction with polyglactin 910 membrane	PD CAL Periapical radiographs	(a) PD (mm) Group A: 6.40 ± 0.32 Group B: 7.25 ± 0.40 (b) CAL (mm) Group A: 6.30 ± 0.40 Group B: 6.50 ± 0.43 (c) Initial defect fill (mm) Group A: 10.73 ± 0.48 Group B: 11.75 ± 0.56	(a) PD (mm)–3 months Group A: 3.75 ± 0.24 Group B: 4.25 ± 0.37 PD (mm)–6 months Group A: 3.05 ± 0.25 Group B: 3.10 ± 0.25 (b) CAL (mm)–3 months Group A: 3.75 ± 0.26 Group B: 3.95 ± 0.47 CAL (mm)–6 months Group A: 2.95 ± 0.23 Group B: 3.20 ± 0.40 (c) Defect fill–3 months (mm) Group A: 8.71 ± 0.44 Group B: 9.41 ± 0.32 Defect fill–6 months (mm) Group A: 8.05 ± 0.44 Group B: 8.68 ± 0.34	Both methods are beneficial for the treatment of infrabony defects.
Veradi. 2019. USA [16]	Retrospective	*n* = 25 Male: 13 Female: 12 Age: 28–58 years (mean age 55.1 ± 10.5 years) NHA in combination with PLGA copolymer	Full mouth plaque score PD CAL Periapical radiographs	(a) PD (mm) 8.32 ± 1.41 (b) CAL (mm) 9.96 ± 1.69	(a) PD–12 months (mm) 4.04 ± 0.84 (b) CAL–12 months (mm) 6.24 ± 1.71 (c) Defect fill–12 months (mm) 4.06 ± 1.66 % Defect fill–12 months 73.3%	NHA in combination with PLGA may give significant improvements for infrabony defects. This case series should not be generalized to larger population.

BOP: bleeding on probing; CAL: clinical attachment level; CPAL: clinical probing attachment level; DFDBA: decalcified freeze-dried bone allograft; FPD: flap procedure for debridement without graft; NHA: nanohydroxyapatite powder; OFD: open flap debridement; PD: probing depth; PLA: polylactic acid granules; PLGA: polylactic acid/polyglycolic acid copolymer.

**Table 2:** Risk of bias assessment of the included studies using STROBE criteria.

Reference	Study Design	Participants	Sample Size	Variable Description	Potential Confounders	Outcome Measurements	Statistical Analysis	Total Score
Yukna [12]	1	1	1	1	1	1	1	7
Meadows [13]	1	1	1	1	1	1	1	7
Prakash [14]	1	0	1	1	1	1	1	5
Chhabra [15]	1	1	1	1	1	1	1	7
Veradi [16]	1	1	1	1	1	1	1	7

## Data Availability

Not applicable.
